# Robust and Accurate Hand–Eye Calibration Method Based on Schur Matric Decomposition

**DOI:** 10.3390/s19204490

**Published:** 2019-10-16

**Authors:** Jinbo Liu, Jinshui Wu, Xin Li

**Affiliations:** Hypervelocity Aerodynamics Institute, Chinese Aerodynamics Research and Development Center, Mianyang 621000, China; wjscardc@126.com (J.W.); lixin_nudt@163.com (X.L.)

**Keywords:** robotics, hand–eye calibration, Schur matric decomposition, observation data preprocessing, outlier detection

## Abstract

To improve the accuracy and robustness of hand–eye calibration, a hand–eye calibration method based on Schur matric decomposition is proposed in this paper. The accuracy of these methods strongly depends on the quality of observation data. Therefore, preprocessing observation data is essential. As with traditional two-step hand–eye calibration methods, we first solve the rotation parameters and then the translation vector can be immediately determined. A general solution was obtained from one observation through Schur matric decomposition and then the degrees of freedom were decreased from three to two. Observation data preprocessing is one of the basic unresolved problems with hand–eye calibration methods. A discriminant equation to delete outliers was deduced based on Schur matric decomposition. Finally, the basic problem of observation data preprocessing was solved using outlier detection, which significantly improved robustness. The proposed method was validated by both simulations and experiments. The results show that the prediction error of rotation and translation was 0.06 arcmin and 1.01 mm respectively, and the proposed method performed much better in outlier detection. A minimal configuration for the unique solution was proven from a new perspective.

## 1. Introduction

The combination of vision sensors and robots is a milestone in robotic intelligence, increasing the extent and efficacy of robot applications [1,2,3,4,5]. Hand–eye calibration is an important technique for bridging the transformation between a robot gripper and a robot vision sensor [6]. Its application is mainly reflected in the robot’s hand–eye coordination, guiding the robot gripper to accurately target and reach into a specified location using the machine vision system. From height work to surgery, the more sophisticated the operation, the better robot hand–eye coordination required.

Many researchers have studied hand–eye calibration, and all current methods can be divided into two categories: linear methods and iterative methods.

Linear methods are efficient and suitable for online hand–eye calibration. Shiu and Ahmad first introduced the dynamic equation *AX* = *XB* into hand–eye calibration and provided minimal configuration for a unique solution [6]. Tsai and Lens proposed a high-efficiency linear method for the equation *AX* = *XB* [7]. Chou and Kamel expressed rotation matrices using quaternions and obtained an analytical solution using Singular Value Decomposition (SVD) [8]. Lu and Chou used an eight-dimension vector to express rotation and translation and obtained a least squares solution [9]. Chen analyzed the relationship between screw movement and hand–eye calibration, and then proved that the movement of the robot gripper and vision sensor must satisfy certain geometric constraints [10]. Daniilidis solved rotation and translation simultaneously by means of a dual quaternion [11]. Park introduced canonical coordinates into the hand–eye calibration equation, which simplified the parameters [12]. Shah constructed a closed-form solution and derived the minimal configuration of the unique solution based on Kronecker product [13]. Compared with Daniilidis [11], Shah’s method was more reliable and accurate. Iterative methods are mainly used to improve the accuracy and robustness. Other authors [14,15] took the F norm of the rotation error and translation error as the cost function, and then optimized it using nonlinear methods. Horaud expressed rotation matrices using quaternions and simultaneously optimized the transformation between the robot-world and hand and eye [16]. Strobl and Hirzinger proposed a new adaptive error model that helped improve the solution to *AX* = *XB* and *AX* = *ZB* [17]. Ruland proposed a self-calibration method that took projection error as its cost function and optimized it using branch-and-bound [18].

The accuracies of the above methods strongly depend on the quality of the observation data. Therefore, preprocessing observation data is essential. Observation data preprocessing is rarely reported. Schmidt et al. [19] proposed a preprocessing method based on vector quantization, which improved the quality of observation data to a certain extent but could not identity outliers. The complexity increased from *O*(*N*) to *O*(*N*^4^), which considerably decreased the method’s efficiency.

## 2. Description of Hand–Eye Calibration Problem

**Figure 1:**
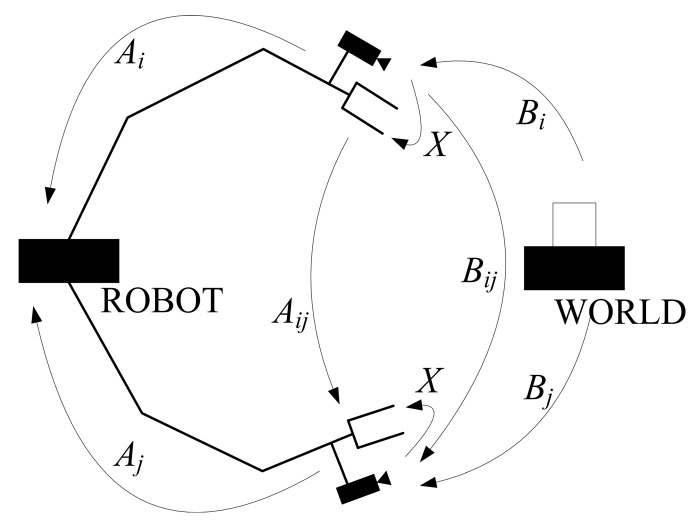
Description of the hand–eye calibration problem.

Figure 1 describes the hand–eye calibration problem. The symbols are notated as follows: *G_i_* is the robot gripper coordinate system, it is fixed on the robot gripper and moves together with it, *C_i_* is the camera coordinate system fixed on the camera that moves together with it and the origin point is coincident with the camera’s optical center. The *Z*-axis is parallel to the optical axis, and the *X* and *Y* axes are parallel to the *X* and *Y* axes of the image coordinate system. *CW* is the world coordinate system and *RW* is the robot coordinate system that is fixed on the robot and moves together with it. When the robot gripper moves, its controlling device can identify the gripper’s pose in *RW*.

*A_i_* is the homogenous transformation matric from *G_i_* to *RW*, obtained from the robot controlling device:

(1) $$A_{i}=\left[\begin{array}{cc} R_{A_{i}} & t_{A_{i}}\\ 0_{1\times 3} & 1 \end{array}\right]$$

*B_i_* is the homogenous transformation matric from *CW* to *C_i_*, obtained using camera pose estimation methods:

(2) $$B_{i}=\left[\begin{array}{cc} R_{B_{i}} & t_{B_{i}}\\ 0_{1\times 3} & 1 \end{array}\right]$$

*A_ij_* is the homogenous transformation matric from *G_i_* to *G_j_*:

(3) $$A_{ij}=A_{j}^{-1}A_{i}=\left[\begin{array}{cc} R_{A_{ij}} & t_{A_{ij}}\\ 0_{1\times 3} & 1 \end{array}\right]$$

*B_ij_* is the homogenous transformation matric from *C_i_* to *C_j_*:

(4) $$B_{ij}=B_{j}B_{i}^{-1}=\left[\begin{array}{cc} R_{B_{ij}} & t_{B_{ij}}\\ 0_{1\times 3} & 1 \end{array}\right]$$

and *X* is the homogenous transformation matric from *C_i_* to *G_i_*:

(5) $$X=\left[\begin{array}{cc} R_{X} & t_{X}\\ 0_{1\times 3} & 1 \end{array}\right]$$

*i* and *j* represent the *i*th and *j*th state of the robot gripper and camera respectively, ranging from 0 to *N*. *N* is the number of movements. Since the robot gripper and camera are fixed, *X* is constant.

The hand–eye calibration equation can be represented by notations:

(6) $$A_{ij}X=XB_{ij}$$

Two equations can be obtained based on the partition matric:

(7) $$\left\{ \begin{array}{l} R_{A_{ij}}R_{X}=R_{X}R_{B_{ij}}\\ \left(R_{A_{ij}}-I\right)t_{X}=R_{X}t_{B_{ij}}-t_{A_{ij}} \end{array}\right.$$

Equation (7) shows that *R_X_* is independent, but the accuracy of *t_X_* is related to *R_X_*.

## 3. Hand–Eye Calibration Method

### 3.1. Schur Matric Decomposition

A given matric can be simplified to a normalized form via similarity transformation. Considering numerical stability, the similarity transformation of a unitary matric is the most attractive. Schur matric decomposition can be simply described as: If *A* ∈ C*^n^*^×*n*^, then a unitary matric that satisfies *U*^H^*AU* = *T* = *D* + *N* exists, where *D* is a diagonal matric and *N* is a strictly upper triangular matric, implicating ∀*I* ≥ *j n_i j_* = 0. For a real matric *A*, *U* is restricted to an orthogonal matric: *U*^T^*AU* = *T*. *T* has the following form:

(8) $$T=\left[\begin{array}{cccc} T_{11} & T_{12} & \cdots & T_{1m}\\ 0 & T_{22} & \cdots & T_{2m}\\ \vdots & \vdots & \ddots & \vdots \\ 0 & 0 & \cdots & T_{mm} \end{array}\right]$$

*T_ii_* is a 1 × 1 or 2 × 2 matric consisting of complex conjugate eigenvalues. If $R_{A_{ij}}$ is similar to $R_{B_{ij}}$ and eigenvalues of $R_{A_{ij}}$ and $R_{B_{ij}}$ are the same, the matric *T* related to $R_{A_{ij}}$ and $R_{B_{ij}}$ are the same.

### 3.2. Hand–Eye Calibration Principle

*A*_0_, *B*_0_ is notated as the initial state of the robot gripper and camera. $\left(A_{i0},{Β}_{i0}\right)\left(i=1,2,\ldots ,N-1,N\right)$ is a series of homogenous transformation matrices related to their initial states. Without the loss of generality, e.g., *i* = 1, only consider the equation related to the rotation in Equation (7):

(9) $$R_{A_{10}}R_{X}=R_{X}R_{B_{10}}$$

From Theorem 1, proved in the Appendix A, the general solution can be written as:

(10) $$R_{X}=U_{R_{A_{10}}}YU_{R_{B_{10}}}^{T}$$

And

(11) $$Y=\left[\begin{array}{ccc} \pm 1 & 0 & 0\\ 0 & c & d\\ 0 & -d & c \end{array}\right],c^{2}+d^{2}=1$$

*R_X_* only depends on *c* and *d*. For arbitrary *i* = 1, 2, …, *N –* 1, *N*:

(12) $$R_{A_{i0}}R_{X}=R_{X}R_{B_{i0}}$$

Substitute Equation (10) into Equation (12):

(13) $$P_{i}Y=YQ_{i}$$

where:

(14) $$P_{i}=U_{R_{A_{10}}}^{T}R_{A_{i0}}U_{R_{A_{10}}},Q_{i}=U_{R_{B_{10}}}^{T}R_{B_{i0}}U_{R_{B_{10}}}$$

Collate Equation (13) into equations only related to *s* = (*c d*)^T^.

(15) $$C_{i}s=D_{i}$$

*C_i_* is a matric generated by the coefficients of *c* and *d*. *D_i_* is a matric generated by the constant term. Then, the final linear equation system can be constructed:

(16) Cs=D

where,

(17) $$C=\left[\begin{array}{c} C_{1}\\ C_{2}\\ \vdots \\ C_{N} \end{array}\right],D=\left[\begin{array}{c} D_{1}\\ D_{2}\\ \vdots \\ D_{N} \end{array}\right]$$

This is a least squares problem with constraints:

(18) $$\begin{array}{l} s=\arg\min\left\{s^{T}Ks-2F^{T}s\right\}\\ s^{T}s=1 \end{array}$$

where,

(19) $$K=C^{T}C,F=C^{T}D,s={\left[\begin{array}{cc} c & d \end{array}\right]}^{T}$$

Notate the cost function as:

(20) $$J\left(s,\lambda \right)=s^{T}Ks-2F^{T}s+\lambda \left(1-s^{T}s\right)$$

From $\frac{\partial J\left(s,\lambda \right)}{\partial s}=0$ and $\frac{\partial J\left(s,\lambda \right)}{\partial \lambda }=0$:

(21) $$\left(K-\lambda I\right)s=F,s^{T}s=1$$

Notate s=(K−λI)y and substitute it into previous equations:

(22) $${\left(K-\lambda I\right)}^{2}y=F$$

*K* is a symmetrical matric, so

(23) $$s^{T}s=y^{T}{\left(K-\lambda I\right)}^{T}\left(K-\lambda I\right)y=y^{T}F$$

*s^T^s* = 1 is the same as *y^T^F* = 1:

(24) $$F=Fy^{T}F=FF^{T}y$$

Because *y^T^F* = *F^T^y*:

(25) $$\left[\lambda^{2}I-2\lambda K+\left(K^{2}-FF^{T}\right)\right]y=0$$

This is a symmetrical second eigenvalue problem [20].

Solve the least squares solution of the Langrage multiplier through methods previously published [21,22]. The least square solution of *s* is:

(26) $$s={\left(K-\lambda_{min}I\right)}^{-1}F$$

Under the condition $\left(U_{R_{A_{10}}},U_{R_{B_{10}}}\right)$, the least squares solution of *R_X_* is:

(27) $$R_{X}^{1}=U_{R_{A_{10}}}Y\left(s\right)U_{R_{B_{10}}}^{T}$$

An $R_{X}^{i}$ exists for each *i* = 1, 2, …, *N* – 1, *N*. To weaken the effect of noise, fuse the matrices based on the string distance of matrices. First, calculate the singular decomposition of the sum of $R_{X}^{i}$, *i* = 1, 2, …, *N* – 1, *N*:

(28) $$U_{R}D_{R}V_{R}^{T}=R_{X}^{1}+R_{X}^{2}+\ldots +R_{X}^{i}+\ldots +R_{X}^{N-1}+R_{X}^{N}$$

Then:

(29) $$R_{X}=U_{R}V_{R}^{T}$$

To solve for *t_X_*, for the *i*th movement, the translation satisfies the following equation:

(30) $$\left(R_{A_{i0}}-I\right)t_{X}=R_{X}t_{B_{i0}}-t_{A_{i0}}\;(i=1,2,\ldots ,N-1,N)$$

Substitute Equation (29) into Equation (30):

(31) $$H_{i}t_{X}=W_{i}$$

Then, a large linear equation system can be obtained:

(32) $$Ht_{X}=W$$

where,

(33) $$\begin{array}{l} H={\left[\begin{array}{ccccccc} H_{1}^{T} & H_{2}^{T} & \ldots & H_{i}^{T} & \ldots & H_{N-1}^{T} & H_{N}^{T} \end{array}\right]}^{T}\\ W={\left[\begin{array}{ccccccc} W_{1}^{T} & W_{2}^{T} & \ldots & W_{i}^{T} & \ldots & W_{N-1}^{T} & W_{N}^{T} \end{array}\right]}^{T} \end{array}$$

This problem can be solved using the least squares method [20].

### 3.3. Outlier Detection

In practice, matrices *A_i_* and *B_i_* contain an observation error, notated as ${\hat{A}}_{i}$ and ${\hat{B}}_{i}$, respectively. *B_i_* is more sensitive to image noises. A poor environment may lead to a large observation error and, in this case, the global optimization solution has no significance. This is a basic problem that considerably decreases the robustness of hand–eye calibration and has not been well solved.

The form of *Y* is:

(34) $$Y=\left[\begin{array}{ccc} \pm 1 & 0 & 0\\ 0 & c & d\\ 0 & -d & c \end{array}\right]$$

$R_{A_{i0}}$ and $R_{B_{i0}}$ must satisfy Equation (13).

(35) $$P_{i}=\left[\begin{array}{ccc} P_{i}^{11} & P_{i}^{12} & P_{i}^{13}\\ P_{i}^{21} & P_{i}^{22} & P_{i}^{23}\\ P_{i}^{31} & P_{i}^{32} & P_{i}^{33} \end{array}\right],\;Q_{i}=\left[\begin{array}{ccc} Q_{i}^{11} & Q_{i}^{12} & Q_{i}^{13}\\ Q_{i}^{21} & Q_{i}^{22} & Q_{i}^{23}\\ Q_{i}^{31} & Q_{i}^{32} & Q_{i}^{33} \end{array}\right]$$

**Figure 2:**
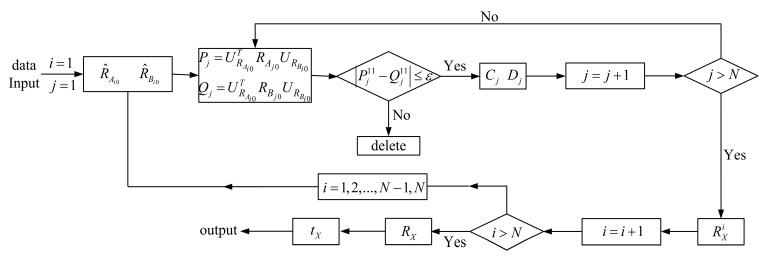
Flowchart of the proposed method.

For arbitrary *c* and *d*, Equation (36) is satisfied:

(36) $$\left|P_{i}^{11}-Q_{i}^{11}\right|\leq \varepsilon$$

which can be used to discriminate the quality of the observation data: if greater than a specific threshold *ε*, then the observation data are outliers and should be deleted. The threshold *ε* is an empirical value. Through setting its value, the observation data can be filtered. The lower the threshold *ε,* the higher the quality of the observation data. In simulations and experiments, *ε* was set to 0.01. In summary, the flowchart of the proposed method is described in Figure 2.

### 3.4. Unique Solution Conditions

Assume the rotation matrices of two movements are *A*_1_, *A*_2_, *B*_1_, and *B*_2_, and *X* is known. From Theorem 1 (Appendix A), the general solution of *A*_1_*X* = *XB*_1_ is:

(37) $$X=U_{A_{1}}YU_{B_{1}}^{T}$$

where, *Y* is a matric only related to *c* and *d.* Substitute Equation (37) into the equation built by two movements:

(38) $$A_{1}X=XB_{1},A_{2}X=XB_{2}$$

Substitute them into Equation (13) to obtain:

(39) $$\left\{ \begin{array}{l} P_{1}Y=YQ_{1}\\ P_{2}Y=YQ_{2} \end{array}\right.$$

And

(40) $$P_{1}=Q_{1}$$

For Equation (39):

(41) $$rank\left(C_{1}^{T}C_{1}\right)=rank\left(\left[\begin{array}{cc} C_{1}^{T}C_{1} & C_{1}^{T}D_{1} \end{array}\right]\right)=0$$

Equation (41) is an identical equation. 

If rotation axes of two movements are not parallel, *P*_2_ and *Q*_2_ are independent:

(42) $$rank\left(C_{2}^{T}C_{2}\right)=rank\left(\left[\begin{array}{cc} C_{2}^{T}C_{2} & C_{2}^{T}D_{2} \end{array}\right]\right)=2$$

From Theorem 2 proved in the Appendix A, if the rotation axes of *N* movements of the robot gripper are parallel, there will be multiple solutions to the hand–eye calibration. Therefore, the minimal configuration of the unique solution is that the robot gripper and camera move at least twice, and the rotation axes cannot be parallel.

## 4. Results

### 4.1. Simulations

We designed simulations to test the performance of different hand–eye calibration methods. The hand–eye calibration equation can be written as:

(43) $$A_{ij}X=XB_{ij}$$

where, *A_ij_* and *B_ij_* are the movement of the robot gripper and camera from time *i* to time *j*, respectively. *A_i_* and *B_i_* were simulated as the observation data. *X* is simulated as the transformation from the camera to the robot gripper. *A_i_*, *B_i_* and *X* consist of rotation matrices and translation vectors. The rotation matric can be generated using three Euler angles.

The simulations included three parts: analysis of noise sensitivity, relationship between the number of movements and accuracy, and outlier detection ability. All the simulations were performed using MATLAB. In addition to the proposed method, we selected another five popular methods for comparisons [7,11,12,13,23]. For the *i*th simulation, ${\tilde{R}}_{X}^{i}$ and ${\tilde{t}}_{X}^{i}$ are the ideal transformation from the camera to the robot gripper and ${\hat{R}}_{X}^{i}$ and ${\hat{t}}_{X}^{i}$ are the measured transformations. The error matric can be calculated as:

(44) $$\begin{array}{l} R_{error}^{i}={\left({\hat{R}}_{X}^{i}\right)}^{T}{\tilde{R}}_{X}^{i}\\ t_{error}^{i}={\hat{t}}_{X}^{i}-{\tilde{t}}_{X}^{i} \end{array}$$

where, $k_{error}^{i}$ and $\theta_{error}^{i}$ are the rotation axis and rotation angle of $R_{error}^{i}$, respectively.

(45) $$\left(k_{error}^{i},\theta_{error}^{i}\right)=rodrigues\left(R_{error}^{i}\right)$$

The errors of rotation and translation are defined as:

(46) $$\begin{array}{l} \theta_{error}=RMS\left(\theta_{error}^{1},\theta_{error}^{2},\ldots ,\theta_{error}^{n-1},\theta_{error}^{n}\right)\\ t_{error}=RMS\left(t_{error}^{1},t_{error}^{2},\ldots ,t_{error}^{n-1},t_{error}^{n}\right) \end{array}$$

where, *n* is the number of simulations.

#### 4.1.1. Analysis of Noise Sensitivity

**Figure 3:**
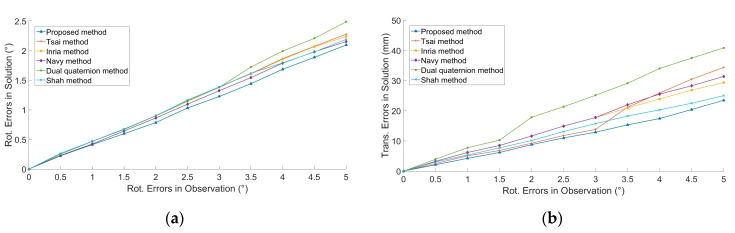

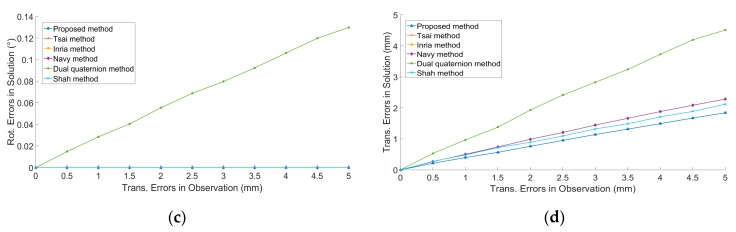
The relationship between calibration accuracy and observation errors: (**a**) Rotation errors in observations and calibration errors of *R_X_*. (**b**) Rotation errors in observations and calibration errors of *t_X_*. (**c**) Translation errors in observations and calibration errors of *R_X_*. (**d**) Translation errors in observation and calibration errors of *t_X_*. Each point on the figure is the Root Mean Square (RMS) of 100 simulations.

Gaussian rotation noise (*μ_R_* = 0, *σ_R_* = 0°–5°) and translation noise (*μ_T_* = 0, *σ_T_* = 0–5 mm) were added into *A_i_* and *B_i_* (*i =* 1, 2, …, 9, 10). We ran 100 simulations at each noise level. The results were shown in Figure 3, in which ‘Rot.’ represents ‘Rotation’ and ‘Trans.’ represents ‘Translation’. Except for the dual quaternion method, translation perturbation had no effect on the rotation solution, because only the dual quaternion method solves rotation and translation simultaneously, whereas other methods solve rotation and translation by steps.

#### 4.1.2. Relationship between Number of Movements and Accuracy

**Figure 4:**
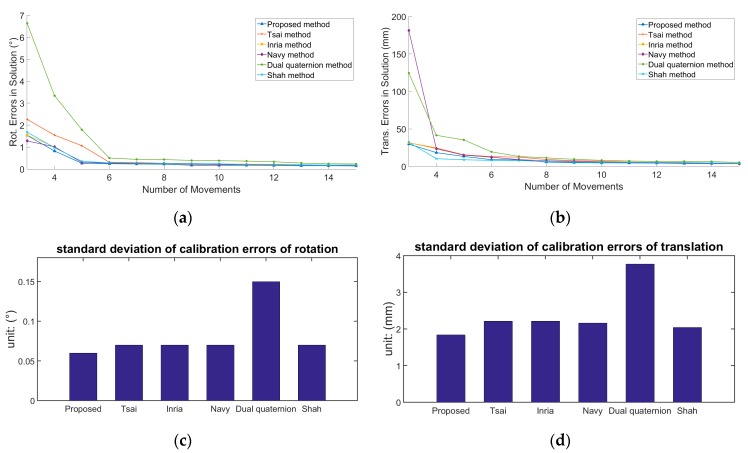
The relationship between calibration accuracy and the number of movements: (**a**) The number of movements and the calibration errors of *R_X_*. (**b**) The number of movements and the calibration errors of *t_X_*. (**c**) The standard deviations of the calibration errors of *R_X_*. (**d**) The standard deviations of the calibration errors of *t_X_*. Each point on the figure is the RMS of 100 simulations.

The simulation conditions included *σ_R_* = 0.2°, *σ_T_* = 2 mm, and the number of movements varied from 3 to 15. We ran 100 simulations at each number of movements. Figure 4a,b indicates that the accuracy of hand–eye calibration improves with the increase in the number of movements. When the number of movements increases from three to eight, the accuracy of hand–eye calibration improves considerably. Figure 4c,d demonstrates that the other five methods are more robust, except for the dual quaternion method being unstable.

#### 4.1.3. Outlier Detection

**Figure 5:**
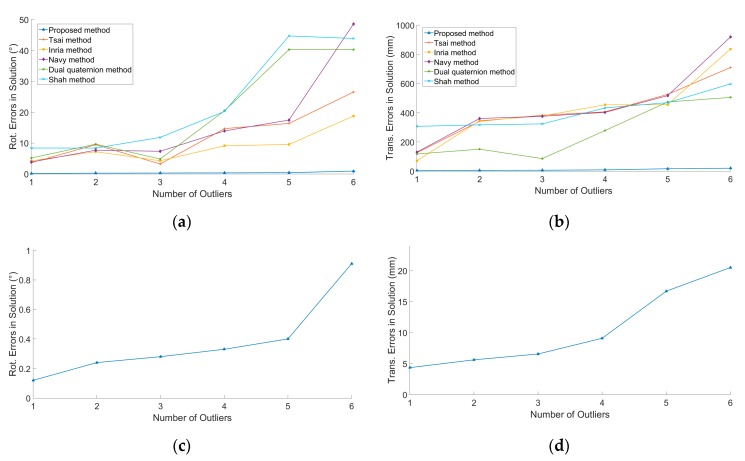
The relationship between calibration accuracy and the number of outliers: (**a**) Calibration errors of *R_X_* and the number of outliers. (**b**) Calibration errors of *t_X_* and the number of outliers. (**c**) Partial enlargers of (**a**). (**d**) Partial enlargers of (**b**). Each point on the figure is the RMS of 100 simulations.

The simulation conditions were *σ_R_* = 0.2°, *σ_T_* = 2 mm, and *ε* = 0.01. The robot gripper moved 10 times, in which large noise was added into *n* (*n* = 1, 2, 3, 4, 5, 6) movements randomly and these observations were regarded as outliers. We ran 100 simulations at each number of outliers. Figure 5a,b shows the relationship between calibration errors of *R_X_* and *t_X_* and the number of outliers, respectively. Figure 5c,d depicts the performance of the proposed method. The results indicate that the proposed method can detect outliers effectively and performs much better than the other five methods.

### 4.2. Experiments

**Figure 6:**
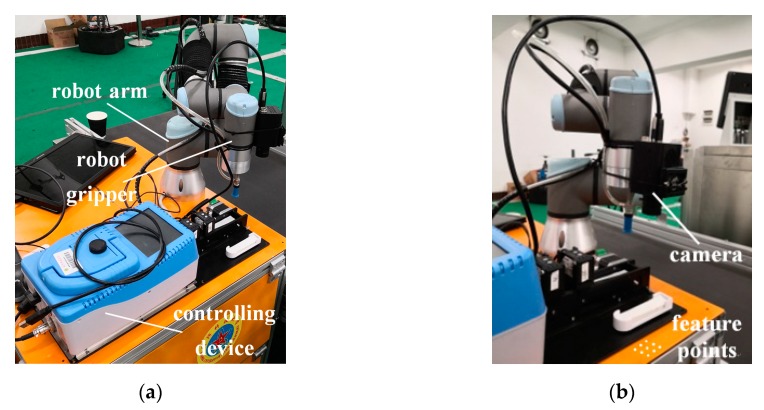
(**a**) Robot arm, gripper and its controlling device. (**b**) Camera and feature points.

Determining poses of the robot gripper with high precision is costly, but movements of the robot gripper can be measured precisely. Thus, most researchers adopt the following program to validate hand–eye calibration methods: the camera moves *N* + *n* times, where the preview *N* times are called the calibration link and the last *n* times are called the verification link. The calibration link is used to solve the transformation between the robot gripper and the camera. The verification link is used to verify method accuracy by comparing its predicted movements with its true movements [3]. The predicted movements of the robot gripper can be solved from the camera’s movements using Equation (47). The true movements of the robot gripper can be obtained from its controlling device. A robot arm was fixed with a camera, as shown in Figure 6a.

For the calibration link: 

(1) Fix 9 feature points on the platform as shown in Figure 6b. The three-dimensional (3D) coordinates of feature points can be measured by Leica Total Station. All the feature points’ coordinates remain unchanged during the experiment.

(2) At time 0, capture an image of the feature points on the platform. Calculate the camera’s pose *B*_0_ through Perspective-n-Points (PnP) methods. The robot gripper’s pose *A*_0_ can be determined from its controlling device.

(3) At time *i*, move the robot gripper and camera.

(4) Capture an image of the feature points on the platform. Calculate the camera’s pose *B_i_* through PnP methods. The robot gripper’s pose *A_i_* can be determined from its controlling device.

(5) Repeat step (3)–(4) *N* times and (*A_i_*_0_, *B_i_*_0_) (*i =* 1, …, *N*–1, *N*) can be obtained.

(6) The transformation *X* from the camera to the robot gripper can be calibrated using all six hand–eye calibration methods.

For the verification link:

(7) Repeat step (3)–(4) *n* times and (*A_i_*_0_, *B_i_*_0_) (*i = N*+1, …, *N*+*n*–1, *N*+*n*) can be obtained.

(8) The predicted movement ${\hat{A}}_{i0}$ of the robot gripper can be calculated through Equation (47). The true movement of the robot gripper *A_i_*_0_ can be obtained from its controlling device.

(47) $${\hat{A}}_{i0}=XB_{i0}X^{-1}$$

(9) Comparing ${\hat{A}}_{i0}$ with *A_i_*_0_, the error matric can be calculated using Equation (48):

(48) $$\begin{array}{l} R_{error}^{i}={\left({\hat{R}}_{A_{i0}}\right)}^{T}R_{A_{i0}}\\ t_{error}^{i}={\hat{t}}_{A_{i0}}-t_{A_{i0}} \end{array}$$

$k_{error}^{i}$ and $\theta_{error}^{i}$ are the corresponding rotation axis and rotation angle of $R_{error}^{i}$, respectively:

(49) $$\left(k_{error}^{i},\theta_{error}^{i}\right)=rodrigues\left(R_{error}^{i}\right)$$

The rotation and translation errors are defined as:

(50) $$\begin{array}{l} \theta_{error}=RMS\left(\theta_{error}^{1},\theta_{error}^{2},\ldots ,\theta_{error}^{n-1},\theta_{error}^{n}\right)\\ t_{error}=RMS\left(t_{error}^{1},t_{error}^{2},\ldots ,t_{error}^{n-1},t_{error}^{n}\right) \end{array}$$

The rotation error is in arcmin and the translation error is in mm.

In the experiment, *N =* 2–9 and *n* = 200. The results are shown in Table 1. The experiment results indicate that the prediction error decreased with the increase in the number of movements and when the robot gripper moved 9 times, the proposed method’s prediction accuracy of rotation exceeded 6 arcsec, which is much higher than the calibration accuracy in the simulations. The reason is explained in the following.

Expand Equation (47) using a partition matric:

(51) $${\hat{R}}_{A_{i0}}=R_{X}R_{B_{i0}}R_{X}^{-1}$$

The prediction error consists of hand–eye calibration error and camera pose estimation error. Hand–eye calibration error is notated as ∆*R_X_*. Then, the prediction error of Equation (51) can be written as:

(52) $$e={\left\|R_{X}\Delta R_{X}R_{B_{i0}}\Delta R_{X}^{-1}R_{X}^{-1}-R_{X}R_{B_{i0}}R_{X}^{-1}\right\|}_{F}\leq \lambda {\left\|\Delta R_{X}R_{B_{i0}}\Delta R_{X}^{-1}-R_{B_{i0}}\right\|}_{F}$$

Equation (52) can weaken the effect of the hand–eye calibration error. This conclusion also applies to the prediction error of translation. Thus, the prediction error in the experiment was much lower than the hand–eye calibration error in the simulations.

## 5. Conclusions

A hand–eye calibration method with high accuracy and robustness was proposed in this paper. Using this method, the basic problem of observation data preprocessing is solved by outlier detection, which significantly improves robustness. However, two aspects remain to be studied. To improve the method’s efficiency, we used the least squares optimization method with constraints. If no strict need exists for efficiency, an iterative method could be considered. We decreased the rotation matric’s dimension from three to two via Schur matric decomposition and unknown parameters satisfied the constraint *c*^2^ + *d*^2^ = 1. If the following triangle transformation is adopted, the degrees of freedom (DOFs) can be decreased from two to one. The Gröbner basis method can be used to solve polynomial equations [24]:

(53) $$c=\frac{2\tan\frac{\theta }{2}}{1+\tan^{2}\frac{\theta }{2}},d=\frac{1-\tan^{2}\frac{\theta }{2}}{1+\tan^{2}\frac{\theta }{2}}$$

## Figures and Tables

**Table 1:** Prediction error: *t_error_* in mm and *θ_error_* in arcmin.

*N*	Proposed		Tsai		Inria		Navy		Dual Quaternion		Shah	
	*θ_error_*	*t_error_*	*θ_error_*	*t_error_*	*θ_error_*	*t_error_*	*θ_error_*	*t_error_*	*θ_error_*	*t_error_*	*θ_error_*	*t_error_*
2	10.14	5.49	10.14	7.06	10.14	6.23	10.17	5.25	10.21	8.70	10.14	5.63
3	10.10	4.63	10.10	6.21	10.14	6.20	10.14	5.10	10.14	7.08	10.10	4.71
4	10.07	4.06	10.10	5.77	10.10	4.74	10.10	4.97	10.14	6.18	10.10	4.16
5	9.83	3.94	10.07	4.15	10.07	3.79	10.07	4.62	10.10	4.17	9.86	4.04
6	0.96	2.46	0.96	3.67	1.30	3.61	2.16	3.54	3.81	3.98	1.34	2.60
7	0.44	1.57	0.51	3.51	0.51	3.60	0.51	1.87	1.78	3.64	0.72	1.75
8	0.37	1.15	0.37	2.76	0.41	2.51	0.44	1.77	0.44	2.27	0.37	1.20
9	0.06	1.01	0.27	2.47	0.34	2.27	0.41	1.19	0.41	1.82	0.20	1.05

## Appendix A

**Lemma** **1.**
*A is a 3 × 3 rotation matric and can be decomposed to Equation (55) based on Schur matric decomposition:*

(54) $$A=U_{A}T_{A}U_{A}^{T}$$

*Then, T_A_ can be written as:*

(55) $$T_{A}=\left[\begin{array}{cc} 1 & 0_{1\times 2}\\ 0_{2\times 1} & T_{2\times 2} \end{array}\right]$$

*and* $T_{2\times 2}$ *is a unit orthogonal matric:*

(56) $$T_{2\times 2}=\left(\begin{array}{cc} a & -b\\ b & a \end{array}\right)$$

**Proof** **of** **Lemma 1.** Because *A* is a unit orthogonal matric:

(57) $$AA^{T}=U_{A}T_{A}T_{A}^{T}U_{A}^{T}=I\Rightarrow T_{A}T_{A}^{T}=I$$

*T_A_* can be written as:

(58) $$T_{A}=\left[\begin{array}{cc} 1 & T_{1\times 2}\\ 0 & T_{2\times 2} \end{array}\right]$$

Substitute *T_A_* into $T_{A}T_{A}^{T}=I$:

(59) $$\left\{ \begin{array}{l} 1+T_{1\times 2}T_{1\times 2}^{T}=1\Rightarrow T_{1\times 2}=\left[\begin{array}{cc} 0 & 0 \end{array}\right]\\ T_{2\times 2}T_{2\times 2}^{T}=I \end{array}\right.$$

Thus, $T_{2\times 2}$ is a unit orthogonal matric. Notate:

(60) $$T_{2\times 2}=\left[\begin{array}{cc} a & c\\ b & d \end{array}\right]$$

Then,

(61) $$\left\{ \begin{array}{l} a^{2}+c^{2}=1\\ b^{2}+d^{2}=1\\ ab+cd=0\\ ad-bc=1 \end{array}\right.\Rightarrow {\left(a-d\right)}^{2}+{\left(b+c\right)}^{2}=0$$

Thus,

(62) $$\left\{ \begin{array}{l} a=d\\ b=-c \end{array}\right.$$

The lemma has been proven. □

**Lemma** **2.**
*For rotation matric A, B, C, D, and X:*

(63) $$\left\{ \begin{array}{l} AX=XB\\ CX=XD \end{array}\right.$$

*and the Schur matric decompositions of A and B are as follows:*

(64) $$\left\{ \begin{array}{l} A=U_{A}T_{A}U_{A}^{T}\\ B=U_{B}T_{B}U_{B}^{T} \end{array}\right.$$

*If the axis of A and B is parallel to the axis of C and D respectively, then:*

(65) $$U_{A}^{T}CU_{A}=U_{B}^{T}DU_{B}=M$$

*The form of M can be written as:*

(66) $$M=\left[\begin{array}{cc} 1 & 0\\ 0 & M_{2\times 2} \end{array}\right]$$

**Proof** **of** **Lemma 2.** *U_A_* and *U_B_* can be obtained by Schur matric decomposition. *k_a_* and *k_b_* are the axes of *A* and *B* calculated through Rodrigues, respectively. *θ_a_* and *θ_b_* are the rotation angles. Then *k_a_* = *Xk_b_*, *θ_a_* = *θ_b_*, *k_c_* = *Xk_d_* and *θ_c_* = *θ_d_*. Then, the rotation matrices can be written as:

(67) $$\left\{ \begin{array}{l} A=\left(1-\cos\theta_{a}\right)E_{A}^{1}+\cos\theta_{a}E_{A}^{2}+\sin\theta_{a}E_{A}^{3}\\ B=\left(1-\cos\theta_{a}\right)E_{B}^{1}+\cos\theta_{a}E_{B}^{2}+\sin\theta_{a}E_{B}^{3}\\ C=\left(1-\cos\theta_{c}\right)E_{A}^{1}+\cos\theta_{c}E_{A}^{2}+\sin\theta_{c}E_{A}^{3}\\ D=\left(1-\cos\theta_{c}\right)E_{B}^{1}+\cos\theta_{c}E_{B}^{2}+\sin\theta_{c}E_{B}^{3} \end{array}\right.$$

$E_{A}^{i}$ and $E_{B}^{i}\left(i=1,2,3\right)$ are linearly independent matrices generated from rotation axes. Any orthogonal transformation has no effect on the property of independence:

(68) $$\left\{ \begin{array}{l} U_{A}^{T}AU_{A}=\left(1-\cos\theta_{a}\right)U_{A}^{T}E_{A}^{1}U_{A}+\cos\theta_{a}U_{A}^{T}E_{A}^{2}U_{A}+\sin\theta_{a}U_{A}^{T}E_{A}^{3}U_{A}\\ U_{B}^{T}BU_{B}=\left(1-\cos\theta_{a}\right)U_{B}^{T}E_{B}^{1}U_{B}+\cos\theta_{a}U_{B}^{T}E_{B}^{2}U_{B}+\sin\theta_{a}U_{B}^{T}E_{B}^{3}U_{B}\\ U_{A}^{T}CU_{A}=\left(1-\cos\theta_{c}\right)U_{A}^{T}E_{A}^{1}U_{A}+\cos\theta_{c}U_{A}^{T}E_{A}^{2}U_{A}+\sin\theta_{c}U_{A}^{T}E_{A}^{3}U_{A}\\ U_{B}^{T}DU_{B}=\left(1-\cos\theta_{c}\right)U_{B}^{T}E_{B}^{1}U_{B}+\cos\theta_{c}U_{B}^{T}E_{B}^{2}U_{B}+\sin\theta_{c}U_{B}^{T}E_{B}^{3}U_{B} \end{array}\right.$$

Because $U_{A}^{T}AU_{A}=U_{B}^{T}BU_{B}=T$,

(69) $$U_{A}^{T}E_{A}^{i}U_{A}=U_{B}^{T}E_{B}^{i}U_{B}\left(i=1,2,3\right)$$

The rotation angles of *C* and *D* are equal, then:

(70) $$U_{A}^{T}CU_{A}=U_{B}^{T}DU_{B}$$

Since,

(71) $$\left\{ \begin{array}{l} U_{A}^{T}CU_{A}T=U_{A}^{T}CAU_{A}\\ TU_{A}^{T}CU_{A}=U_{A}^{T}ACU_{A} \end{array}\right.$$

and the axes of *A* and *C* are parallel,

(72) AC=CA

Thus,

(73) $$U_{A}^{T}CU_{A}T=TU_{A}^{T}CU_{A}\Rightarrow MT=TM$$

From Lemma 1, *M* can be written as:

(74) $$M=\left[\begin{array}{cc} 1 & 0\\ 0 & M_{2\times 2} \end{array}\right]$$

*M*_22_ is a unit orthogonal matric and Lemma 2 has been proven. □

**Theorem** **1.**
*A, X and B are 3 × 3 rotation matrices and AX = XB. The Schur decomposition of A and B can be written as:*

(75) $$\left\{ \begin{array}{l} A=U_{A}T_{A}U_{A}^{T}\\ B=U_{B}T_{B}U_{B}^{T} \end{array}\right.$$

*Notate*$Y=U_{A}^{T}XU_{B}$. *Then,*

(76) $$Y=\left(\begin{array}{ccc} \pm 1 & 0 & 0\\ 0 & c & d\\ 0 & -d & c \end{array}\right),\;c^{2}+d^{2}=1$$

**Proof** **of** **Theorem 1.** Since *A* is similar to *B*, $T_{A}=T_{B}=T$. Substitute it into *AX* = *XB*, then:

(77) $$U_{A}TU_{A}^{T}X=XU_{B}TU_{B}^{T}\Rightarrow TY=YT$$

From Lemma 1, *T* can be obtained:

(78) $$T=\left[\begin{array}{cc} 1 & 0\\ 0 & T_{2\times 2} \end{array}\right]$$

Assume $Y=\left[\begin{array}{cc} Y_{1\times 1} & Y_{1\times 2}\\ Y_{2\times 1} & Y_{2\times 2} \end{array}\right]$, then:

(79) $$\left\{ \begin{array}{l} Y_{1\times 1}=Y_{1\times 1}\\ Y_{1\times 2}\left(T_{2\times 2}-I\right)\equiv 0\\ \left(T_{2\times 2}-I\right)Y_{2\times 1}\equiv 0\\ T_{2\times 2}Y_{2\times 2}\equiv Y_{2\times 2}T_{2\times 2} \end{array}\right.$$

Due to arbitrariness,

(80) $$\begin{array}{l} Y_{1\times 2}=\left[\begin{array}{cc} 0 & 0 \end{array}\right]\\ Y_{2\times 1}={\left[\begin{array}{cc} 0 & 0 \end{array}\right]}^{T} \end{array}$$

Thus,

(81) $$Y=\left[\begin{array}{cc} Y_{1\times 1} & 0_{1\times 2}\\ 0_{2\times 1} & Y_{2\times 2} \end{array}\right]$$

Assume

(82) $$T_{2\times 2}=\left[\begin{array}{cc} a & b\\ -b & a \end{array}\right],\;Y_{2\times 2}=\left[\begin{array}{cc} c & e\\ d & f \end{array}\right]$$

then:

(83) $$\left\{ \begin{array}{l} ac+bd\equiv ac-be\Rightarrow d=-e\\ ae+bf\equiv bc+ae\Rightarrow f=c\\ -bc+ad\equiv ad-bf\Rightarrow f=c\\ -be+af\equiv bd+af\Rightarrow d=-e \end{array}\right.$$

*Y* is a unit orthogonal matric, so:

(84) $$\left\{ \begin{array}{l} c^{2}+d^{2}=1\\ Y_{1\times 1}=\pm 1 \end{array}\right.$$

The ± of *Y*_11_ is related to the determinant of *U_A_U_B_*. Because the determinant of *X* is greater than 0, the symbol of *Y*_11_ is the same as the symbol of the determinant of *U_A_U_B_*. Theorem 1 has been proven. □

**Theorem** **2.**
*If rotation axes of N movements of robot gripper are parallel, there will be multiple solutions to the hand–eye calibration.*

**Proof** **of** **Theorem 2**. Assume rotation matrices of two movements are *A_1_, A_2_, B_1_,* and *B_2_. X* is an unknown rotation matric. From Theorem 1, the general solution of equation *A_1_X = XB_1_* can be obtained:

(85) $$X=U_{A_{1}}YU_{B_{1}}^{T}$$

*Y* is a matric only related to *c* and *d:*

(86) $$Y=\left[\begin{array}{ccc} \pm 1 & 0 & 0\\ 0 & c & d\\ 0 & -d & c \end{array}\right]$$

Substitute the general solution into the second movement:

(87) $$A_{2}U_{A_{1}}YU_{B_{1}}^{T}=U_{A_{1}}YU_{B_{1}}^{T}B_{2}\Rightarrow U_{A_{1}}^{T}A_{2}U_{A_{1}}Y=YU_{B_{1}}^{T}B_{2}U_{B_{1}}$$

From Lemma 2:

(88) $$U_{A_{1}}^{T}A_{2}U_{A_{1}}=U_{B_{1}}^{T}B_{2}U_{B_{1}}=M=\left[\begin{array}{cc} 1 & 0_{1\times 2}\\ 0_{2\times 1} & M_{2\times 2} \end{array}\right]$$

Thus,

(89) MY≡YM

The equation is an identical equation indicating that the second movement cannot provide any extra constraint related to *c* and *d*. In the same way, an *N* – 1 movement with same rotation axes cannot provide any extra constraint related to *c* and *d*. The general solution applies to all equations built by *N* movements. Therefore, hand–eye calibration problems with same rotation axes have multiple solutions. Theorem 2 has been proven. □
